# Artemether-lumefantrine versus artemisinin-naphthoquine in Papua New Guinean children with uncomplicated malaria: a six months post-treatment follow-up study

**DOI:** 10.1186/s12936-015-0624-4

**Published:** 2015-03-21

**Authors:** Moses Laman, John M Benjamin, Brioni R Moore, Mary Salib, Somoyang Tawat, Wendy A Davis, Peter M Siba, Leanne J Robinson, Timothy ME Davis

**Affiliations:** School of Medicine and Pharmacology, University of Western Australia, Fremantle Hospital, PO Box 480, Fremantle, 6959 WA Australia; Papua New Guinea Institute of Medical Research, Madang, Papua New Guinea; Infection and Immunity Division, Walter and Eliza Hall Institute, Parkville, VIC Australia; Department of Medical Biology, University of Melbourne, Melbourne, VIC Australia

**Keywords:** Malaria, *Plasmodium falciparum*, *Plasmodium vivax*, Artemisinin combination therapy, Naphthoquine, Effectiveness

## Abstract

**Background:**

In a recent trial of artemisinin-naphthoquine (artemisinin-NQ) and artemether-lumefantrine (AM-LM) therapy in young children from Papua New Guinea (PNG), there were no treatment failures in artemisinin-NQ-treated children with *Plasmodium falciparum* or *Plasmodium vivax* compared with 2.2% and 30.0%, respectively, in AM-LM-treated children during 42 days of follow-up. To determine whether, consistent with the long elimination half-life of NQ, this difference in efficacy would be more durable, clinical episodes of malaria were assessed in a subset of trial patients followed for six months post-treatment.

**Methods:**

For children completing trial procedures and who were assessable at six months, all within-trial and subsequent clinical malaria episodes were ascertained, the latter by clinic attendances and/or review of hand-held health records. Presentations with non-malarial illness were also recorded. Differences between allocated treatments for pre-specified endpoints were determined using Kaplan-Meier survival analysis.

**Results:**

Of 247 children who were followed to Day 42, 176 (71.3%) were included in the present sub-study, 87 allocated to AM-LM and 89 to artemisinin-NQ. Twenty children in the AM-LM group (32.8%) had a first episode of clinical malaria within six months compared with 10 (16.4%) in the artemisinin-NQ group (*P* = 0.033, log rank test). The median (interquartile range) time to first episode of clinical malaria was 64 (50–146) *vs* 116 (77–130) days, respectively (*P* = 0.20). There were no between-group differences in the incidence of first presentation with non-malarial illness (*P* = 0.31).

**Conclusions:**

The greater effectiveness of artemisinin-NQ over conventional AM-LM extends to at least six months post-treatment for clinical malaria but not non-malarial illness.

**Trial registration:**

Australian New Zealand Clinical Trials Registry ACTRN12610000913077.

## Background

Artemisinin-based combination therapy (ACT) has emerged as first-line treatment for uncomplicated malaria in most malaria-endemic countries because of the high cure rate, relative safety, transmission-blocking potential, and ability to delay the development of parasite drug resistance [1]. Its place in management of malaria has been cemented through randomized clinical trials, but there is uncertainty regarding the optimal duration of follow-up for determining efficacy. The World Health Organization (WHO) currently recommends Day 42 as the maximum follow-up time-point for trials involving longer half-life ACT partner drugs [2,3], but there is theoretical evidence that at least 56 days of follow-up should be considered for trials of falciparum malaria involving ACT partner drugs with elimination half-lives that are longer than one week [4]. In the case of vivax malaria, and in trials of falciparum malaria in areas where *Plasmodium vivax* is transmitted, late post-treatment recurrences can result from relapses from dormant liver stages. Durations of follow-up of up to 63 days have been employed in this situation [5]. However, a longer duration of follow-up increases the complexity and cost of clinical trials, while attrition rates of patients, and also trained research staff [6], are likely to increase.

In a recent comparative efficacy and safety trial of three daily doses of artemisinin-naphthoquine (artemisinin-NQ) and conventional artemether-lumefantrine (AM-LM) therapy in young children from north coastal Papua New Guinea (PNG) with uncomplicated falciparum and/or vivax malaria [7], there were no reappearances of asexual forms of either *Plasmodium falciparum* or *P. vivax* during the WHO-recommended 42 days of follow-up in any child in the artemisinin-NQ group. The greater number of recurrent infections after AM-LM may have reflected the shorter elimination half-life of LM compared with NQ (4–5 days [8] vs 21–25 days [9]). To assess this possibility and in light of the possible need for prolonged follow-up in such ACT trials [4], a subset of trial participants were followed for a period of six months post-treatment. It was hypothesized that, in coastal PNG where malaria transmission is at least moderate and recurrent malaria in children aged ≤5 years is common [10], the incidence of clinical episodes of malaria during prolonged follow-up would reflect the elimination half-life of the non-artemisinin partner drug.

## Methods

### Study setting, design and approvals

The present study was conducted at Mugil and Alexishafen Health Centres in Madang Province on the north coast of mainland PNG. The malaria entomological inoculation rate (EIR) in this setting has been estimated at 37 for *P. falciparum* and 24 for *P. vivax* [10]. Although there has been a significant decline in falciparum malaria after the introduction of malaria control programs, the incidence of vivax malaria has remained unchanged over the last three decades [10,11].

The parent trial was a randomized, comparative, efficacy trial of AM-LM and artemisinin-NQ (Australian New Zealand Clinical Trials Registry ACTRN12610000913077) [7]. In the present sub-study, all children completing trial procedures were followed subsequently for two primary outcomes; i) the cumulative incidence of clinical episodes of malaria within-trial (up to Day 42) and post-trial (up to 6 months after treatment), and ii) the median time to the clinical episodes of malaria within six months of allocated treatment. Secondary outcomes were; i) the incidence of non-malarial illness between treatment groups within six months of allocated treatment, and ii) the median time to episodes of a non-malarial illness within six months of allocated treatment. Both the parent trial and present sub-study were approved by the PNG Institute of Medical Research Review Board, the PNG Medical Research Advisory Committee (MRAC 10.39) and the University of Western Australia Human Research Ethics Committee. Written informed consent was obtained from parents/guardians of all children before participation.

### Patients

Children aged between 6 months and 5 years with uncomplicated malaria were recruited to the parent trial between March 2011 and April 2013 provided that they had; i) an axillary temperature >37.5°C or fever during the previous 24 hours, ii) *P. falciparum* (>1,000 asexual parasites/μL whole blood) and/or *P. vivax* (>250/μL) on a peripheral blood smear, iii) not used study drugs in the past 14 days, and iv) no clinical or laboratory evidence of severe malaria or other infection [7].

### Within-trial clinical and laboratory procedures

Within-trial clinical and laboratory procedures have been described previously [7]. Briefly, children were randomly assigned by use of a computer-generated random numbers in blocks of 24 by site to receive AM-LM (1.7:10 mg/kg; Novartis Pharma, Basel, Switzerland) twice-daily for three days with milk as per manufacturer’s recommendation or artemisinin-NQ (20:8 mg/kg; Kunming Pharmaceutical Corporation, Yunnan, China) daily with water for three days. Treatment allocation was concealed from investigators by use of an opaque, sealed, and sequentially numbered envelope that was opened only after the patient was enrolled. Serial clinical and laboratory assessments including axillary temperature and malaria microscopy were performed on Days 1, 2, 3, 7, 14, 28 and 42.

Early treatment failure was defined as the development of signs of severity or an inadequate parasitological response by Day 3 [12]. Any child who developed parasitaemia between Days 4 and 42 was considered to have late parasitological failure or, if febrile (axillary temperature >37.3°C), late clinical failure. Children without any of the above WHO definitions of treatment failure were considered to have an adequate clinical and parasitological response (ACPR). *Plasmodium falciparum* re-infection and recrudescence were distinguished using polymerase chain reaction genotyping of merozoite surface proteins (MSP1 and MSP2) based on paired samples collected on Day 0 and at reappearance during the active follow-up period [13,14]. *Plasmodium vivax* recrudescence was determined based on genotyping of MSP1F3 and Microsatellite 16 [15]. Malaria blood smears were examined independently by two skilled microscopists [7]. The WHO-recommended method of parasite density calculation based on the number/200-500 leucocytes and an assumed leucocyte count of 8,000/μL [16] was used and has been validated for the study setting [17].

### Post-trial follow-up

During the post-trial follow-up period, parents/guardians were asked to bring their children back to the Mugil or Alexishafen Health Centres if the child became unwell or developed fever within the six months after allocated treatment. In all these cases, field microscopy for malaria parasites or a malaria rapid diagnostic test (RDT; CareStart® HRP-2/pLDH (Pf/pan) Combo Test, AccessBio, Somerset, NJ, USA) was performed. All malarial and non-malarial illnesses during the post-trial follow-up period were treated according to PNG national guidelines [18].

Initially, the parents/guardians of the children who did not re-attend with an illness within six months were visited in their villages at the end of this period and their child’s health record books were examined to document malarial or non-malarial illnesses that may have been missed due to attendance at another health care facility. However, this practice was discontinued because of logistic difficulties and the fact that an additional primary endpoint was identified in less than <1% of these cases. However, those children still within the six-month follow-up period when the Mugil and Alexishafen Health Centres closed (on Day 42 of the last randomized patient) were excluded from the present study because all their post-trial outcomes could not be ascertained.

### Statistical analysis

Data are presented as mean ± SD or median [inter-quartile range]. Two-sample comparisons for normally-distributed variables were by Student’s *t*-test, for non-normally distributed variables by Mann Whitney U-test, and for proportions by Fisher’s exact test. Differences between allocated treatments for pre-specified primary and secondary endpoints were determined using Kaplan-Meier survival analysis and the log rank test. All *P*-values are two-tailed and unadjusted for multiple comparisons.

## Results

### Patients

**Figure 1:**
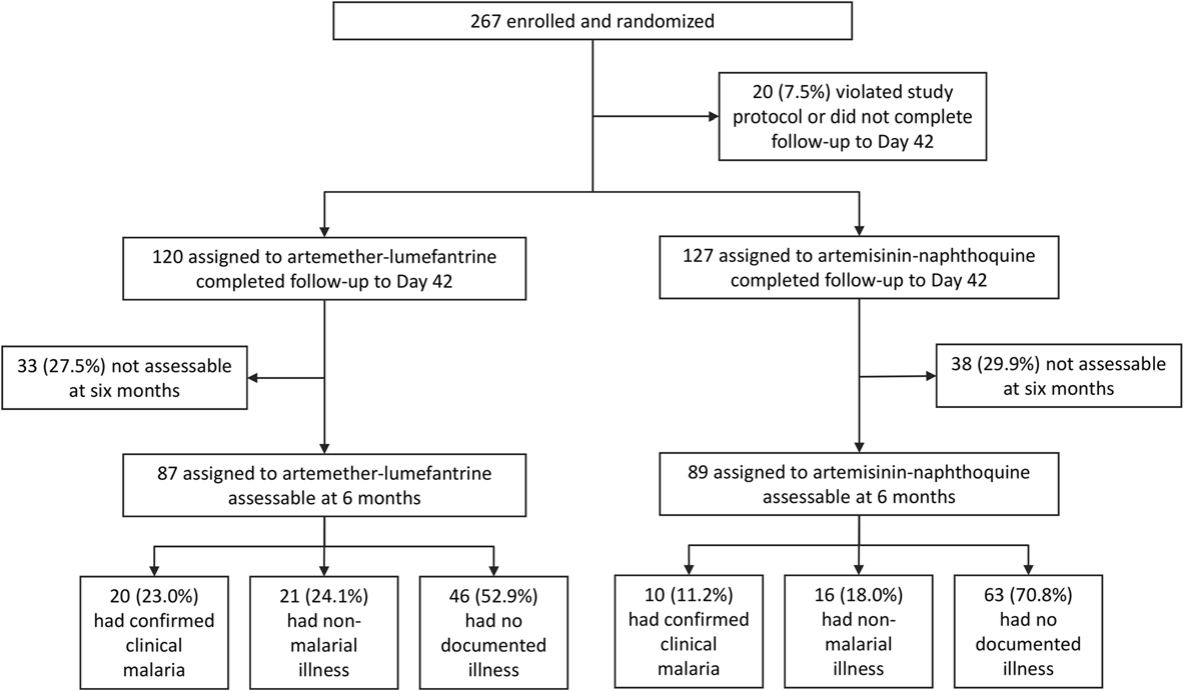
**Profile of patients followed from randomization to the end of the six-month follow-up period.**

Of the 267 children randomized in the parent trial, 247 (92.5%) were followed to Day 42. Of this latter number, 176 (71.3%) were included in the six month analysis, comprising 87 allocated to AM-LM and 89 to artemisinin-NQ (see Figure 1). The 71 children who were excluded were those who had not attended the Mugil and Alexishafen Health Centres post-trial and were still within the six month follow-up period when the two clinics closed at the end of the parent trial. There were no deaths or non-infection-related clinic attendances amongst the 176 included children, and no differences in baseline characteristics by allocated treatment except that more artemisinin-NQ-treated children presented with vivax malaria (see Table 1).

**Table 1:** **Baseline characteristics of children who completed six months of follow-up classified by allocated treatment**

	**Artemether-lumefantrine (n = 87)**	**Artemisinin-naphthoquine (n = 89)**	***P*** **-value**
Age (months)	41.4 ± 16.6	44.0 ± 15.4	0.28
Male (%)	43 (49.4)	47 (52.8)	0.76
Falciparum malaria (%)*	80 (92.0)	73 (82.0)	0.07
Vivax malaria (%)*	10 (11.5)	21 (23.6)	0.04
Axillary temperature (°C)	38.3 ± 1.3	38.1 ± 1.4	0.51
Weight (kg)	12.4 ± 2.5	12.6 ± 2.5	0.50
Respiratory rate (/min)	31.1 ± 8.8	31.3 ± 10.1	0.97
Pulse rate (/min)	122.8 ± 20.8	122.5 ± 20.7	0.91
Mid upper arm circumference (cm)	14.5 ± 1.2	14.5 ± 1.2	0.97
Height (cm)	93.1 ± 10.6	93.0 ± 10.5	0.97
Body Mass Index (kg/m^2^)	14.4 ± 1.6	14.7 ± 2.1	0.38
Palpable spleen ≥2 cm (%)	35 (40.2)	38 (42.7)	0.76
Haemoglobin (g/L)	89.8 ± 17.1	94.0 ± 17.7	0.11
Blood glucose (mmol/L)	6.6 ± 1.8	6.7 ± 1.8	0.71

Data are numbers (percentages) or mean ± SD.

*8 patients (3 artemether-lumefantrine-treated and 5 artemisinin-naphthoquine-treated) had mixed *P. falciparum*/*P. vivax* infections.

### Episodes of clinical malaria

**Figure 2:**
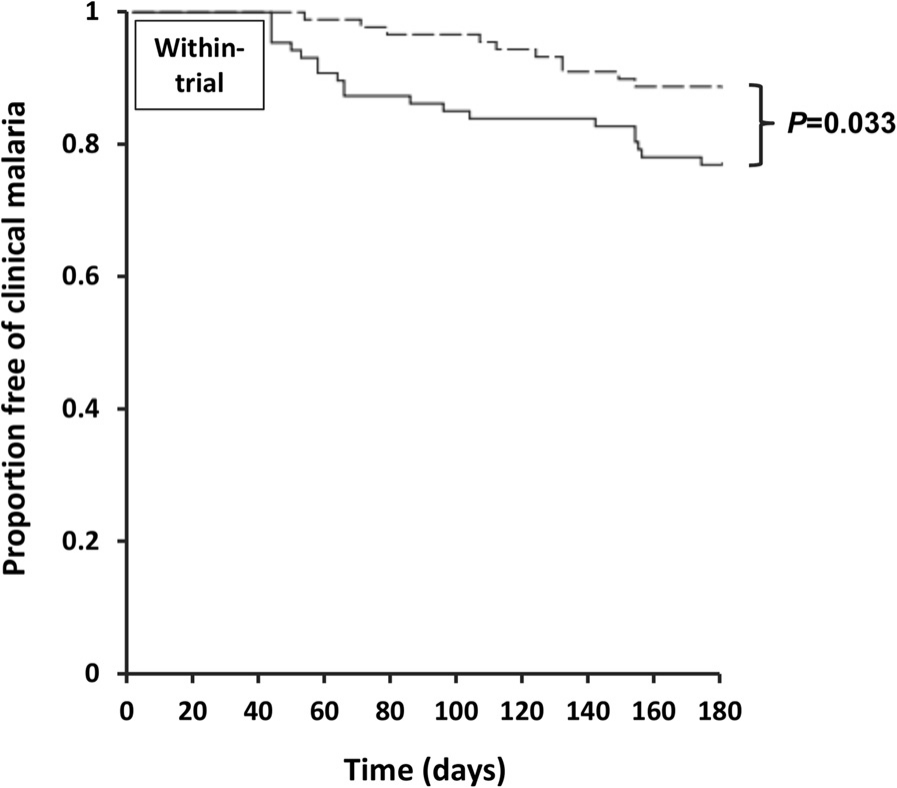
**Kaplan-Meier survival analysis showing the proportions of children treated with artemether-lumefantrine (solid line) and artemisinin-naphthoquine (dashed line) remaining free of clinical malaria during six months after allocated treatment.** The *P*-value for the log rank test is shown.

In the AM-LM group, 20 (32.8%) children had a first episode of clinical malaria (due to either *P. falciparum* or *P. vivax*) within six months of treatment (see Table 2). This included 4 who developed clinical malaria within-trial [7], 12 who developed clinical malaria post-trial, and 4 who were recorded as late parasitological failure during the trial without being treated and who subsequently developed clinical malaria during the post-trial period on Days 56, 62, 140 and 154, respectively. Of these 20 patients, 18 (90%) had falciparum malaria and two (10%) had vivax malaria at enrolment. In the artemisinin-NQ group, there was no late parasitological or clinical failure during the 42 days within-trial. However, 10 children developed clinical malaria between Days 52 and 152 (16.4%, *P* = 0.046 vs AM-LM; see Table 2). Of these 10 patients, five (50%) had falciparum malaria, four (40%) had vivax malaria and 1 (10%) had a mixed species infection at baseline. Kaplan-Meier analysis showed that the incidence of a first episode of clinical malaria during six months of follow-up was higher in the AM-LM group (*P* = 0.033 by log rank test; see Figure 2). The median time to the first episode of clinical malaria was 64 days in the AM-LM and 116 days in the artemisinin-NQ group (*P* = 0.20; see Table 2).

**Table 2:** **Endpoints by allocated treatment for the 176 children with data for six months after trial entry**

	**Artemether-lumefantrine (n = 87)**	**Artemisinin-naphthoquine (n = 89)**	***P*** **-value**
Primary outcome			
Clinical malaria within six months following initial treatment	20/87 (23)	10/89 (11.2)	0.046
Median time to first malarial illness (days)	64 [50–146]	116 [77–130]	0.20
Secondary outcome			
Incidence of non-malarial illnesses within six months	21/87 (24.1)	16/89 (18.0)	0.36
Median time to first non-malarial illness (days)	123 [80–149]	107 [92–166]	0.77

Data are number (percentage) or median [interquartile range].

If only those children who had an ACPR at Day 42 in the parent trial were considered, 12 of the 37 in the AM-LM group (32.4%) and 10 of 89 in the artemisinin-NQ group (11.2%) had an episode of clinical malaria between six weeks and six months after treatment (*P* = 0.008).

From the available data relating to species of *Plasmodium* detected in the 30 patients who developed clinical malaria during follow-up, these children were classified as belonging to one of five sub-groups; i) definite *P. falciparum* (slide or RDT positive for this species), ii) definite *P. vivax* (slide or RDT positive for this species), iii) definite mixed *P. falciparum/P.vivax* (slide positive for these species), iv) *P. falciparum* with or without *P. vivax* (an RDT reported as ‘mixed’ since the presence of both HRP-2 and pan-pLDH lines indicates an infection with *P. falciparum*, or a mixed infection including *P. falciparum*), and v) unknown (RDT recorded as positive without further details in the health record book). There was no difference between the proportions of children in each category by allocated treatment (see Table 3; *P* = 0.12 by Fisher’s exact test). In 19 out of the 30 cases (63.3%), there was definite slide or RDT evidence of *P. falciparum* infection.

**Table 3:** ***Plasmodium*** **species categorization of cases of clinical malaria during follow-up, based on microscopy and/or rapid diagnostic testing, by allocated treatment**

**Category**	**Artemether-lumefantrine (n = 20)**	**Artemisinin-naphthoquine (n = 10)**
Definite *P. falciparum*	2 (10%)	3 (30%)
Definite *P. vivax*	3 (15%)	0
Definite mixed species infection	0	1 (10%)
*P. falciparum* ± *P. vivax*	9 (45%)	4 (40%)
Unknown	6 (30%)	2 (20%)

### Episodes of non-malarial illness

Twenty-one (24.1%) and 16 (18.0%) of children in the AM-LM and artemisinin-NQ groups, respectively, had a first episode of non-malarial illness during the six-month follow-up period (*P* = 0.36; see Table 1). Kaplan-Meier analysis showed that the incidence of non-malarial illness was not significantly different between groups (*P* = 0.31 by log rank test). There was also no significant between-group difference in the time to the first episode of a non-malarial illness (medians 123 and 107 days, respectively; see Table 2). Most had respiratory infections (32.4%) or skin infections (24.3%). There were no differences in the percentages of children who had more than one non-malarial illness during follow-up (63.2% in AM-LM group vs 55.1% in the artemisinin-NQ group; *P* = 0.27).

## Discussion

The present study shows that the relative clinical benefits of artemisinin-NQ over conventional AM-LM therapy extend well beyond the 42 days of the parent trial. Artemisinin-NQ was associated with approximately half the risk of a first episode of clinical malaria during follow-up over six months. The parallel nature of the Kaplan-Meier curves and the between-treatment difference in median time to first malarial episode suggest that the long half-life of the NQ component of artemisinin-NQ delays malaria post-treatment susceptibility relative to AM-LM by 7–8 weeks. This is equivalent to 2–3 NQ elimination half-lives in PNG children [9]. In the patients allocated to AM-LM, the relatively rapid elimination of LM and its active metabolite desbutyl-LM [8,19] is unlikely to afford protection from reappearance of malaria or a new malarial infection beyond the 42 days of the parent trial. Despite the higher haemoglobin concentrations in the artemisinin-NQ group at the end of the parent trial [7] and the recognized association between anaemia and bacterial infection in young children in the tropics [20-22], there was no protective effect of artemisinin-NQ treatment on the risk of non-malarial (mainly bacterial) infections in patients in this study.

There is evidence that local malaria transmission intensity may play a role in the efficacy of longer-acting partner drugs [23,24]. In one trial in which patients were followed up beyond Day 42 in an area of intense malaria transmission in Uganda (*P. falciparum* EIR 562 [25]), there was no difference in malaria reinfection rates in children receiving AM-LM or dihydroartemisinin-piperaquine at Day 63 despite the longer elimination half-life of piperaquine [23]. In the more moderate transmission setting of the present study (*P. falciparum* EIR 37 [10]), the post-treatment prophylactic effect of NQ was evident at Day 63 and beyond. However, the ability of NQ to suppress relapses of *P. vivax* may have been a contributor to the between-group differences in late clinical malaria in the patients [7], a consideration not relevant to sub-Saharan Africa. Unfortunately, the exact relative contribution of *P. vivax* infections could not be ascertained accurately because in 70% of cases the RDT was reported as ‘mixed’ or simply positive.

Antimalarial drugs such as NQ with long elimination half-lives are more likely to promote the development of parasite resistance as a result of prolonged sub-therapeutic plasma concentrations [26,27]. In the case of PNG, chloroquine has provided a salutary example in that sustained and widespread use has resulted in clinically significant parasite resistance and the need for alternative treatments [28]. However, the ‘mandatory’ combination of newer long-acting drugs such as NQ with an artemisinin derivative [1,3] is likely to be protective, and even triple therapy regimens are being considered to limit the possibility of parasite resistance [29]. The widespread deployment of artemisinin-NQ in countries such as PNG may be justifiable based on prolonged efficacy/effectiveness such as demonstrated in the parent trial [7] and present data, but *in vivo*, *in vitro* and/or genetic monitoring of parasite resistance should be an integral part of this process.

The present study had limitations. Neither accurate differentiation between *Plasmodium* species nor genotyping to differentiate recrudescences from reinfections was performed, but the primary endpoint (clinical malaria) was a pragmatic one designed to allow the long-term real-world effectiveness of the two treatment regimens to be assessed. For logistic and financial reasons, not all participants in the parent trial at each site were followed for the full six months, but most children were included and the two treatment groups were similar in number and baseline characteristics. Indeed, the significantly greater number of vivax cases in the artemisinin-NQ group and the potentially high rate of associated late relapses may have led to bias against this ACT, but it nonetheless proved more effective than AM-LM.

## Conclusion

The present study is one of the few published in the literature that have employed long-term follow-up of patients with malaria treated with artemisinin combination therapy. The data suggest strongly that the long terminal elimination half-life of three daily doses of NQ confers benefit of artemisinin-NQ over AM-LM to at least six months post-treatment. This observation should favourably influence cost-effectiveness analyses. In addition, the present long-term follow-up data also add to the reassuring results of detailed safety monitoring over 42 days in the parent trial [7], in that there were no unsuspected late sequelae after three daily doses of artemisinin-NQ rather than the single dose recommended by the manufacturer.

## Abbreviations

ACPR: Adequate clinical and parasitological response
ACT: Artemisinin combination therapy
AM: Artemether
EIR: Entomological inoculation rate
LM: Lumefantrine
MSP: Merozoite surface protein
NQ: Naphthoquine
PNG: Papua New Guinea
WHO: World Health Organization
